# Difficult-to-Treat Spondyloarthritis in Morocco: A Real-World Study

**DOI:** 10.31138/mjr.290124.dtt

**Published:** 2024-12-31

**Authors:** Salma Zemrani, Bouchra Amine, Imane ElBinoune, Samira Rostom, Rachid Bahiri

**Affiliations:** Department of Rheumatology A, El Ayachi Hospital, University Hospital Centre Ibn Sina, Rabat-Sale, Morocco

**Keywords:** axial spondyloarthritis, difficult-to-treat, biological therapy

## Abstract

**Objectives:**

High biologic requirement in inflammatory rheumatic diseases (IRD) may indicate a difficult to treat (D2T) condition. In axial spondyloarthritis (axSpA), a consensual definition for this concept is still lacking. Our objectives are to identify the prevalence and characteristics of multiswitcher patients with axSpA, and to analyse the number and reasons for switches.

**Methods:**

This is a longitudinal observational study including patients treated with biologic agents for axSpA. We propose to define D2T patients as those who required more than 2 b/tsDMARD. Patients who did not fulfil this definition were used as controls. The prevalence of multiswitchers was calculated, and characteristics were compared between the two groups. The number and reasons for switches were analysed in the D2T group.

**Results:**

124 patients were included. The prevalence of multiswitchers was 24.19%. There were no significant differences between the two groups in the age, sex, and comorbidities. D2T patients have more arthritis (p=0.01), and fibromyalgia (p=0.04), and higher disease activity before initiating biotherapy, (BASDAI:p=0.04), (ASDAS:p=0.04). Additionally, the time from diagnosis to the first use of biologic was longer (p=0.04). In the multivariate analysis, the D2T condition was found to be associated with fibromyalgia (p=0.01). Among this group, the prevalence of those treated with 3, 4, and 5 b/tsDMARD was 86%, 9%, and 5%, respectively, the primary and secondary failures were the most common reasons for switching.

**Conclusion:**

We suggest that D2T-axSpA present several characteristics. Identification of this category in large studies is necessary to establish a consensus definition.

## INTRODUCTION

A relatively new and noteworthy concept in the realm of rheumatic diseases, specifically rheumatoid arthritis (RA), is the difficult-to-treat condition (D2T).^1^ In 2021, the EULAR (European Alliance of Associations for Rheumatology) task force developed criteria for identifying D2T RA. This includes persistent symptoms even after the failure of at least two biologic agents or targeted synthetic disease-modifying antirheumatic drugs (b/tsDMARDs) with varying mechanisms of action.^2^ The task force also outlined points to consider for managing patients falling into this category.^3^

This concept of D2T is emerging in the field of rheumatology and has been discussed in other diseases, including spondyloarthritis (SpA). However, a consensus definition has not yet been established.^4^

AxSpA is known for its diverse presentation, including both radiographic and non-radiographic axial involvement, peripheral manifestations such as arthritis, enthesitis, and dactylitis, and the potential for extra-musculoskeletal manifestations like uveitis, psoriasis, and inflammatory bowel disease. These varied manifestations pose challenges in both diagnosis and treatment strategies.^5^

In recent years, the treatment of axSpA has advanced significantly due to various factors including a more precise comprehension of pathophysiology, the emergence of novel classes of targeted therapies, and the development of novel strategies like treat to target (T2T) and tight control.^6,7^ As a result, it has been proposed that treating patients to achieve remission or low disease activity, is an achievable objective.^8^ However, there are still many cases where some patients require frequent treatment rotations for various reasons such as lack of efficacy and adverse events.^9^ This scenario may indicate a D2T disease.

Few studies in the literature have described this multiple switching in routine care and tried to outline the contributing factors. In Morocco, spondyloarthritis is still associated with a significant delay in diagnosis and a high prevalence of active and severe forms. In addition, the unavailability of some biologic agents and the lack of a global social insurance for all patients could make the disease difficult to manage.

Since there is currently no consensus on the definition of D2T axSpA, our study hypothesised that multiple switching (requirement of > 2 biologics/ts DMARD) may define this situation. Our primary objective was to investigate the prevalence of D2T axSpA in routine practice and the secondary objectives were to identify associated factors with our local D2T axSpA as well as the phenotypic differences compared to non-D2T group, and to analyse the circumstances of switching.

## METHODS

### Patients

This was a retrospective longitudinal observational study conducted at a tertiary rheumatology centre. Patients diagnosed with axSpA based on the ASAS (Assessment of spondyloarthritis international society) classification,^10^ and who had continuous follow-up in our centre since January 2014 were included if they started their first b/ts DMARD treatment at least 12 months before the beginning of study. The data of these patients were reviewed, starting from their initial visits until their last visits.

### Data Collection

We collected demographic features, comorbidities, age at time of axSpA diagnosis, disease duration, disease characteristics including phenotypic presentation, radiographic involvement and extra-musculoskeletal manifestations.

BASDAI (Bath Ankylosing Spondylitis Disease Activity Index), ASDAS CRP (Ankylosing Spondylitis Disease Activity Score - CRP) and BASFI (Bath ankylosing spondylitis functional index) were used to assess disease activity and functional impairment prior to the initial biologic treatment and at the last visit.

Information about previous and current uses of b/tsDMARDs, as well as the time from diagnosis to the first use of b/tsDMARDs were also collected.

### Definition of D2T axSpA

Due to the lack of consensus on the concept of difficult-to-treat spondyloarthritis, we hypothesised that multiple switches between biologics may define this situation. Therefore, we propose defining D2T patients as those who require more than two biologics/tsDMARDs (D2T Group).

### Characteristics of D2T axSpA

The demographic data, comorbidities, disease characteristics, disease activity prior to bDMARD initiation and at the final visit, as well as prior biologic use, were compared between the D2T group and the control group, which included patients who did not fulfil the proposed definition of D2T axSpA (non-D2T group).

Furthermore, the factors associated with the D2T group were identified through univariate and multivariate analysis.

### Reason for switches in D2T group

In a subsequent phase, the number of switches between biologics in the D2T group and the reasons for these switches were analysed.

### Statistical Analysis

Statistical analysis was performed using SPSS software, version 13.0. Normally distributed continuous variables were presented as mean ± standard deviation (SD), and asymmetric variables were expressed as median ± interquartile range (IQR defined as 25–75th percentiles). Qualitative data were presented as frequencies (number and percentage). The comparisons between D2T and non-D2T groups were examined using the T student and Mann-Whitney tests for quantitative variables and using the Chi-squared test or Fischer’s exact test for qualitative variables. p values less than 0.05 were considered statistically significant.

Univariate and multivariate analysis were conducted to determine associated factors to D2T patients.

## RESULTS

### Baseline characteristics

One hundred twenty-four patients were included in our study. Baseline characteristics are summarised in **Table 1**. 65.6% of the patients were male. The mean age at diagnosis was 38.15±11.8 years-old, and the mean disease duration was 12.59±7.27 years. The mean body mass index (BMI) was 25.23±2.25 kg/m^2^. 36.9% had an ongoing or history of smoking, and 12.6% had associated fibromyalgia. Arthritis was found in 55% of patients, and enthesitis in 12.9%. Concerning extra-musculoskeletal manifestations, a history of uveitis was found in 12.9%, psoriasis in 4%, and inflammatory bowel disease in 13.7%. 49.2 % had coxitis. In terms of disease activity, mean BASDAI, ASDAS CRP, and BASFI before first biotherapy were 4.62±1.38, 3.62± 0.91, and 5.02±2.21, respectively (**Table 2**).

**Table 1.** Baseline characteristics of patients in the D2T axSpA and the non–D2T axSpA groups.

	**Total (124)**	**D2TaxSpA D2T 30 (24.19%)**	**non-D2T axSpA 94 (75.8%)**	**p**
**Age (year)^1^**	38.15±11.8	39.83±12.59	37.62 ±11.55	0.37
**Sex (male)^2^**	81(65.6%)	15 (50%)	66 (70.2%)	0.09
**BMI (kg/m2)^1^**	25.23 ± 2.25	24.94 ± 1.53	25.31 ± 2.76	0.46
**Smoking^2^**	38 (36.9%)	9 (42.9%)	29 (35.4%)	0.52
**Hypertension^2^**	10 (8.1%)	3 (10%)	7 (7.4%)	0.65
**Diabetes^2^**	7 (5.6%)	2 (6.7%)	5 (5.3%)	0.82
**Fibromyalgia^2^**	16 (12.9%)	9 (30%)	7 (7.4%)	0.01
**Age of diagnosis (year)^1^**	28.63±11.78	27.37±10.33	29±12.2	0.53
**Disease duration (year)^1^**	12.59 ±7.27	14.35 ±6.91	12.09 ±7.33	0.16
**Arthritis^2^**	69 (55%)	22 (73.3%)	47(50%)	0.02
**Enthesitis^2^**	16 (12.9%)	4 (13.3%)	12 (12.8%)	0.93
**Radiographic sacroiliitis^2^**	111 (89.5%)	26 (86.7%)	85 (90.4%)	0.55
**Non-Radiographic sacroiliitis^2^**	13 (10.5%)	4 (13.3%)	9 (9.6%)	0.28
**Uveitis^2^**	16 (12.9%)	5 (16.7%)	11 (11.7%)	0.48
**Psoriasis^2^**	5(4%)	0	5 (5.3%)	0.19
**IBD^2^**	17 (13.7%)	4 (13.3%)	13 (13.8%)	0.94
**Coxitis^2^**	61 (49.2%)	16 (53.3%)	45 (47.9%)	0.6

1 Mean and standard deviation;

2 Number and percentage. IDB: inflammatory bowel disease; BMI: body mass index.

**Table 2.** Disease activity in D2T axSpA and the non-D2T axSpA groups.

	**Total (124)**	**D2TaxSpA D2T 30 (24.19%)**	**non-D2T axSpA 94 (75.8%)**	**p**
ESR (mm/h)^1^*	54.95±27.38	59.79±34.99	53.35±24.38	0.26
CRP (mg/ml)^2^*	31.92 [15.4–59.06]	31.5[22.75–52.5]	29 [12.9–53]	0.02
BASDAI1*	4.62±1.38	5.2±1,47	4.43±1.3	0.008
ASDAS CRP1*	3.62± 0.91	3.93±1.07	3.5±0.82	0.03
BASFI1*	5.02±2.21	5.5±2.34	4.86±2.16	0.18
ESR (mm/h)2**	17 [7–31.5]	20 [8.5–38]	16 [7–30]	0.25
CRP (mg/ml) 2**	6 [2.72–11.8]	11.4 [4.18–24.25]	5.2 [2.5–9.72]	0.01
BASDAI2**	1.31 [0.6–0.2]	2.1 [1.42–3]	1.2 [0.5–1.6]	0.001
ASDAS CRP1**	1.68 ± 0.85	2.52 ± 0.94	1.5 ± 0.7	0.001
BASFI1**	2.93 ± 1.94	3.65 ± 1.56	2.75 ± 1.43	0.01

1 Mean and standard deviation;

2 Median and IQR.

ESR: erythrocyte sedimentation rate; CRP: C-reactive protein; BASDAI: Bath Ankylosing Spondylitis Disease Activity Index; ASDAS CRP: Ankylosing Spondylitis Disease Activity Score – CRP; BASFI: Bath ankylosing spondylitis functional index.

* Disease activity before initiating biotherapy.

** Disease activity in the last visit.

### Prevalence of D2T axSpA

30 (24.19%) patients fulfilled the criteria for D2T axSpA (requirement of >2biologics/tsDMARD).

### Characteristics of D2T axSpA compared with non-D2T axSpA

There were no significant differences between the two groups in the age at disease diagnosis, sex, BMI, smoking history, and comorbidities. Compared to the control group, patients in the D2T group had more arthritis (73.3% versus 50%, p=0.02) and more associated fibromyalgia (30% versus 7.4%, p = 0.01) (**Table 1**).

Regarding disease activity, CRP, BASDAI and ASDAS-CRP before initiating first biologic were higher in the D2T group, (BASDAI: p = 0.008), (ASDAS CRP: p = 0.03, (CRP: p = 0.02). Additionally, activity parameters remain higher among multiswitchers patients at the last visit comparatively to the control group, (CRP: p = 0.01), (BASDAI: p = 0.00), (ASDAS CRP: p = 0.00), (BASFI: p = 0,01) (**Table 2**). The treatment history was also compared between both groups. The time from diagnosis to the first use of biologic treatments was longer in the D2T group: 4 [2.25–10] years versus 3 [1–6] years (p=0.04). In the D2T group, all patients had received Tumour necrosis factor (TNF)-alpha inhibitors, 38.1% had received anti-interleukin 17 (anti-IL17), and only one patient had received Janus Kinase (JAK) Inhibitors (**Table 3**). Furthermore, D2T patients received more than two biologic agents over a duration of 5.29±1.53 years.

**Table 3.** Treatments history in the D2T axSpA and the non-D2T axSpA groups since 2014.

	**Total (124)**	**D2TaxSpA D2T 30 (24.19%)**	**non-D2T axSpA 94 (75.8%)**	**p**
**Delay Diagnosis-First biologic^2^**	1 [1–8]	4 [2.25–10]	3 [1–6]	0.04
**TNFi^1^**	89 (71.77%)	30 (100%)	68 (72.34%)	0.04
**IL17i^1^**	27 (21.77%)	8 (38.1%)	19 (20.21%)	0.16
**JAKi^1^**	1 (0.8%)	1 (3.33%)	0	-

1 Mean and standard deviation;

2 Median and IQR.

TNFi: Tumour necrosis factor-alpha inhibitors; JAKi Janus-kinase inhibitors; IL17i: interleukin 17-inhibitors.

### Factors associated with D2T axSpA

As reported in **Table 4**, fibromyalgia was found to be an associated factor with D2T axSpA (OR, 4.7; 95%CI, 1.41–15.65; P = 0.01) in the multivariate analysis. In the other hand, while an association was observed between D2T axSpA and arthritis (P = 0.02), as well as activity scores before initiating first biologic (BASDAI (P = 0.04), ASDAS CRP (P = 0.03)) in univariate regression analysis, this association did not remain statistically significant in multivariate analysis.

**Table 4.** Associated factors D2T axSpA by univariate and multivariate logistic regression.

**Variable**	**Univariate analysis**			**Multivariate Analysis**						
	OR	95% CI	P	OR	95% CI	P
**Fibromyalgia**	5.32	1.77- 15.94	0.003	4.70	1.41–15.65	0.01
**Arthritis**	2.75	1.11–6.79	0.02	1.74	0.64–4.72	0.27
BASDAI*	2.96	1.04–8.45	0.04	1.39	0.95–2.02	0.08
ASDAS CRP*	1.69	1.03–2.77	0.03	1.43	0.8–2.53	0.21
**Time before first biologic**	1.02	0.96–1.09	0.45	1.03	0.95–1.11	0.45

OR: Odds ratio; CI: Confidence interval; BASDAI: Bath Ankylosing Spondylitis Disease Activity Index; ASDAS CRP: Ankylosing Spondylitis Disease Activity; Score–CRP.

* Activity before initiating biotherapy.

### Number and reasons for switches in D2T group

**Figure 1.**
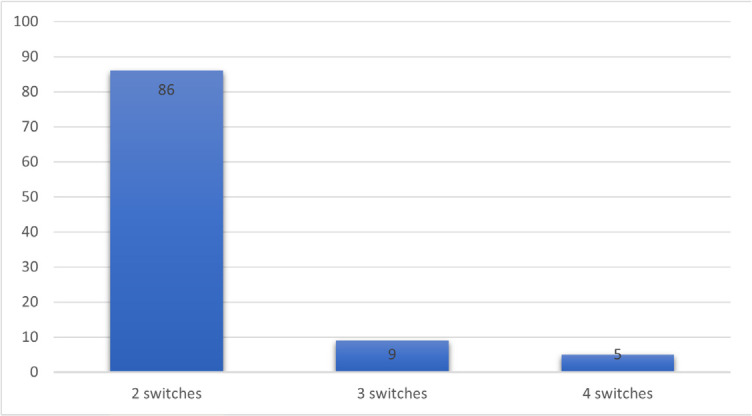
Number of switches in D2T group.

**Figure 2.**
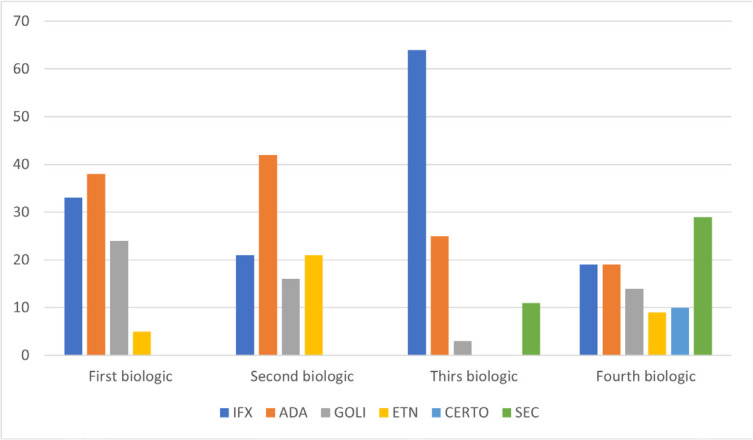
Biologics type requirements during different switches in D2T group. ADA: Adalimumab; IFX: Infliximab; ETN: Etanercept; GOLI: Golimumab; CERTO: Certolizumab; SEC: Secukinumab.

**Figure 3.**
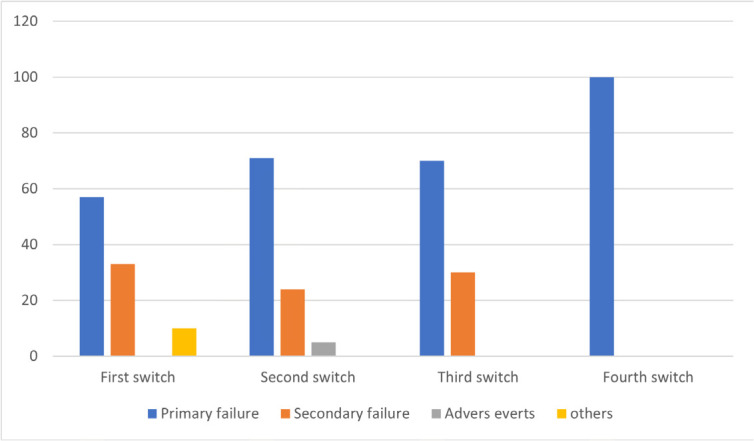
Reasons for switch in D2T group.

The number and reasons for switches, as well as the types of different biologics required in the D2T group are described in **Figures 1, 2, and 3**. Among these patients, the prevalence of those treated with 3, 4, and 5 b/ts DMARD was 86%, 9%, and 5%, respectively. The first switch was related to primary failure in 57% of cases, secondary failure in 33%, and other reasons related to socioeconomic conditions in 10%. In the second switch, primary and secondary failures were noted in 71% and 24% of cases, respectively, with side effects accounting for 5%. Concerning the third switch, 70% of patients presented primary failure and 30% secondary failure. A fourth switch was observed in only one patient due to primary failure.

## DISCUSSION

How could we define D2T axSpA?

To date, there is no consensus on the definition of D2T patients with axSpA. The direct application of D2T-RA criteria in the context of SpA may present several limitations due to significant differences between the two diseases.^11^ Furthermore, in rheumatoid arthritis there are currently several classes of targeted treatment with differing modes of action,^12^ while in axSpA, only three are currently available (anti-TNF, anti-IL17, JAKi).^13^

In routine practice, some patients with axSpA do not respond to several consecutive biologic treatments, due to several reasons such as lack of effectiveness and adverse events,^9^ which may suggest a difficult-to-treat condition. Therefore, in our study, we suggest defining SpA D2T as having a high biologic requirement (>2 biologics/ts DMARD).

### What is the real-life prevalence of this condition?

In our study, 30 patients (24.19%) met the definition of high biologic usage, and this result is concordant with literature data. It is commonly estimated that the prevalence of this condition ranges from 10% to 30%.^14^ In a French study, 19.59 % out of 10798 patients with axSpA were muliswitchers.^9^ In another cohort of 227 patients, high biologic requirement was found to be 27%.^15^ A recent report based on Scandinavian registries including over 6000 patients, found that during a 3-year follow-up, 8%, 3%, and 1% of patients received at least 3, 4, or 5 targeted therapies respectively.^16^ Finally, the study by Phillipoteaux et al. found that 28.3% of patients received more than two biologic agents.^17^

### Which characteristics could define D2T patients?

Our analysis showed that fibromyalgia was significantly associated with D2T axSpA, which is consistent with literature data and in particular the findings of Phillipoteaux et al.^17,18^ In addition, Bello et al. reported that the switching rate was significantly higher in SpA patients treated with TNF-inhibitors with associated fibromyalgia when compared to patients without fibromyalgia (15.2% vs 4%, respectively), furthermore, retention rate of anti-TNF-alpha after 2 years was also shorter in this patient category.^19^

In axSpA, phenotypic presentation may also contribute in therapeutic response. It has showed in a cohort of 305 axSpA, that patients with peripheral manifestations have higher disease activity than patients with purely axSpA.^20^ An analysis of the ASAS-COMOSPA Study also demonstrated that patients with peripheral involvement had higher levels of PRO (patient reported outcome) and greater use of drugs.^21^ Our results are in keep with these studies; indeed, peripheral involvement was founded to be more frequent in D2T group.

Concerning disease activity, frequent use of biologic agents was also associated with high activity scores in our population, BASDAI, CRP and ASDAS-CRP before initiation of the first biologic were more elevated in the D2T group. Accordingly, we suggest that a high inflammatory burden may be a predictive factor for failing to achieve remission and as a result, evolving into a difficult-to-treat disease. In an analysis including patients with spondyloarthritis from the DESIR cohort, it was demonstrated that 5-year remission under TNF-inhibitors was more difficult to achieve in those with higher disease activity at baseline.^22^

Furthermore, a significant time delay from diagnosis to first bDMARDs treatment was found in our D2T group, this finding implies the concept of the window of opportunity suggesting that an early treatment can suppress inflammation and as a consequence prevent irreversible outcomes.^23^ In addition, other multiple factors may also contribute to potential D2T axSpA. As mentioned in the Scandinavian study, comorbidities and extra-musculoskeletal manifestations were found to be associates with multiple treatment switches.^16^

### Reasons for switches

In the second part of our analysis, we examined the reasons for switching between different biologic agents. As shown in **Figure 3**, the first, second and third switches were mostly related to primary and secondary failure, knowing that TNF-inhibitors were the first line treatment in our D2T group, and this finding is consistent with literature data. A review by A. Deodar et al. found that switching due to lack or loss of efficacy was more common than switching due to intolerance.^24^ Additionally, multiple studies showed that patients who switched because of secondary failure or adverse events were significantly more responsive to a second TNF-inhibitor than patients with primary failure. This hypothesis was supported by the clinical trial of Rudwaleit et al. who reported that ASAS40 and BASDAI50 response rates were higher for patients who switched because of loss of response or intolerance than for patients who switched because of primary lack of efficacy.^25^

Concerning therapeutic options, current data on secukinumab does not support its superiority over anti-TNF after previous TNF-inhibitor treatment.^26,27^ This suggests that despite therapeutic advances in spondyloarthritis, patients are still experiencing multiple switches due to lack of efficacy. Data from our study is insufficient to make conclusions about the efficacy of JAK-inhibitors in D2T patients. In the current literature, the SELECT-axis 2 study demonstrated the superior efficacy of Upadacitinib compared to placebo in patients with radiographic axSpA and inadequate response to bDMARDs. However, prior exposure to 2 bDMARDs was noted in only 30% of patients.^28^

From another perspective, our results showed that the first switch was related in 10% to non-adherence due to lack of access to treatment related to socioeconomic circumstances. Non-adherence has been considered a contributing factor to D2T RA in the EULAR definition, and several studies have supported this.^29^ In light of this finding, we suggest that socioeconomic burden may contribute to D2T disease and may be applicable to the D2T spondyloarthritis definition.

### How could we manage D2T patients?

Our study found a significant delay in time from diagnosis to initial bDMARDs treatment in the D2T group. Therefore, we suggest that early intervention is important not only for preventing structural damage but also the onset of a D2T condition. Additionally, assessment of comorbidities that may influence the evaluation of disease, particularly fibromyalgia. is a crucial step to prevent overestimate disease activity.

There is also a need to address non-adherence, which is usually associated with higher levels of disease activity and may lead to inappropriate treatment switching. Several factors contribute to this, including socio-economic conditions, psychological factors, poly-medication and perceptions of lack of efficacy.^30^ Indeed, facilitating access to healthcare system, therapeutic education, and a shared decision-making strategy should be used to optimize compliance with treatments.^31^

Concerning switches strategies, high-quality evidence concerning D2T patients still limited. It is recommended to consider the phenotypical presentation as well as the reason for previous switches when deciding on a biologic agent. Additionally, other therapeutic strategies, such as the combination of two targeted therapies could be evaluated.

Finally, and similarly to RA,^2^ there is a need for consensual recommendation or point to consider managing patients with axSpA with high biologics requirement.

## STRENGTHS AND LIMITS OF THE STUDY

While some recent studies have described the characteristics of difficult-to-treat spondyloarthritis, our study is the first of its kind conducted in North Africa and the Middle East. This geographical focus sets our research apart from previous studies, which may have primarily focused on other regions. This is significant because disease characteristics and treatment patterns can vary across different geographic locations. In addition to describing the characteristics and associated factors of difficult-to-treat spondyloarthritis, our study provides further insight by examining the reasons for treatment switches and the difficulties of access to bDMARDs related to socioeconomic circumstances and lack of global insurance, which could make the disease difficult to manage. However, the study has some limitations. Firstly, there is currently no consensus definition of D2T SpA. Therefore, we have proposed using the number of treatment criteria to define this category of patients. Furthermore, we did not record data concerning depression, and we lacked sufficient information on radiographic progression in our patients.

In conclusion, our study indicates that multiple switches between biologic agents due to ineffectiveness and adverse events may be a defining criterion for D2T axSpA. Furthermore, this is a multifactorial condition with various contributing factors, including the inflammatory burdens of the disease, comorbidities, phenotypic presentation, and socioeconomic conditions. Identifying this category of patients in large cohorts across different countries is necessary to establish a consensus definition and individualized management approach.
